# Statistical methods for modeling repeated measures of maternal environmental exposure biomarkers during pregnancy in association with preterm birth

**DOI:** 10.1186/1476-069X-14-9

**Published:** 2015-01-26

**Authors:** Yin-Hsiu Chen, Kelly K Ferguson, John D Meeker, Thomas F McElrath, Bhramar Mukherjee

**Affiliations:** Department of Biostatistics, University of Michigan School of Public Health, Ann Arbor, MI USA; Department of Environmental Health Sciences, University of Michigan School of Public Health, Ann Arbor, MI USA; Division of Maternal-Fetal Medicine, Brigham and Women’s Hospital, Harvard Medical School, Boston, MA USA

**Keywords:** Prematurity, Environment, Statistical methods, Biomarkers, Repeated measures

## Abstract

**Background:**

It is of critical importance to evaluate the role of environmental chemical exposures in premature birth. While a number of studies investigate this relationship, most utilize single exposure measurements during pregnancy in association with the outcome. The studies with repeated measures of exposure during pregnancy employ primarily cross-sectional analyses that may not be fully leveraging the power and additional information that the data provide.

**Methods:**

We examine 9 statistical methods that may be utilized to estimate the relationship between a longitudinal exposure and a binary, non-time-varying outcome. To exemplify these methods we utilized data from a nested case–control study examining repeated measures of urinary phthalate metabolites during pregnancy in association with preterm birth.

**Results:**

The methods summarized may be useful for: 1) Examining sensitive windows of exposure in association with an outcome; 2) Summarizing repeated measures to estimate the relationship between average exposure and an outcome; 3) Identifying acute exposures that may be relevant to the outcome; and 4) Understanding the contribution of temporal patterns in exposure levels to the outcome of interest. In the study of phthalates, changes in urinary metabolites over pregnancy did not appear to contribute significantly to preterm birth, making summary of average exposure across gestation optimal given the current design.

**Conclusions:**

The methods exemplified may be of great use in future epidemiologic research projects intended to: 1) Elucidate the complex relationships between environmental chemical exposures and preterm birth; 2) Investigate biological mechanisms in prematurity using repeated measures of maternal factors throughout pregnancy; and 3) More generally, address the relationship between a longitudinal predictor and a binary, non-time-varying outcome.

**Electronic supplementary material:**

The online version of this article (doi:10.1186/1476-069X-14-9) contains supplementary material, which is available to authorized users.

## Background

Preterm birth, defined as delivery before 37 weeks completed gestation, is both a significant public health problem and a multifactorial disease [1]. In attempt to identify predictive markers, underlying causes, and/or mechanistic pathways, many research projects have investigated the contribution of various maternal or fetal factors during pregnancy in relation to risk of prematurity. One example is the investigation of biomarkers of inflammation; C-reactive protein, interleukins (e.g., IL-6), matrix metalloproteinases, and angiogenic factors measured in maternal blood, amniotic fluid, or various other matrices during pregnancy have been explored with rigor as predictors of preterm birth [2–4]. Additionally, a number of studies have examined biomarkers of environmental chemical exposures which may be important contributors to prematurity [5].

A majority of these studies have utilized measurements from one time point during pregnancy [5]. However, there is additional utility in having multiple measures across gestation. For markers with poor reproducibility within subject, having multiple measures of exposure may give a more accurate representation of average exposure and confer greater power for detecting an association with preterm birth. For markers with good reproducibility within subject, having multiple measures may provide additional information about the relationship between exposure and disease, if the relationship does vary across time. For example, they could provide information about windows of vulnerability during gestation [6].

Methods for examining data with a longitudinal exposure and non-time-varying outcome do not fall in the standard realm of generalized linear mixed models as instead of having correlated outcome data, the repeated measures of the exposures are correlated. There is no general consensus on how the information contained in the longitudinal exposure trajectory can be used in a binary regression model [7]. Consequently, the few studies with this type of data simply examine a series of cross-sectional associations as well as an average of repeated measures across pregnancy in standard binary regression analyses [8, 9]. This paper illustrates several different methods that may be used to examine this unique but not uncommon data structure, specifically for epidemiologic studies that examine biomarkers during pregnancy, when fewer measurements may be available because of practical limitations in collecting repeated samples from a large number of expecting mothers. We also specify, for data with specific characteristics, which methods may be more useful for powerfully detecting subtle associations or for capturing relationships between exposure profiles over time in relation to a binary endpoint of interest.

## Methods

We applied each method using data from a nested case–control study examining exposure to phthalates during pregnancy and preterm birth. The study population and measurement of phthalate monoester metabolites in urine samples has been described in detail previously [10]. Briefly, mothers were recruited early in pregnancy at Brigham and Women’s Hospital in Boston, MA, as part of a prospective cohort. In addition to demographic information, mothers provided urine samples for phthalate measurement at up to four time points during pregnancy. From this population 130 mothers who delivered preterm and 352 random controls were selected and their urine samples extracted from freezer storage for phthalate analysis. Collection times for urine samples within this study population were median 9.79 for visit 1 (range 4.71 to 16.1), 17.9 for visit 2 (range 14.9 to 21.9), 26.0 for visit 3 (range 22.9 to 29.3) and 35.1 for visit 4 (range 33.1 to 38.3) weeks gestation [11]. The number of phthalate measurements available at each visit was relatively stable for visits 1–3 (visit 1 = 479, visit 2 = 422, visit 3 = 412) but fewer were available at visit 4 (N = 380) as many of the preterm cases had already delivered at that time point [11]. Phthalate metabolites were measured using high performance liquid chromatography and tandem mass spectrometry by NSF International in Ann Arbor, MI [11]. At the time of metabolite measurement, specific gravity was also measured in urine samples as an indicator of urine dilution using a digital handheld refractometer (Atago Co., Ltd., Tokyo, Japan). For the present study, repeated measures of mono-2-ethylhexyl phthalate (MEHP) as well as mono-*n*-butyl phthalate (MBP) were examined as they were both observed to be associated with preterm birth in previously published results [10, 11], but differ in variability of measurements across gestation. Based on intraclass correlation coefficients (ICC), MEHP is less stable over time (ICC = 0.30, 95% confidence interval [CI] = 0.25, 0.35) compared to MBP (ICC = 0.57, 95% CI = 0.53, 0.62) [11]. As with many biomarker measurements, distributions of MEHP and MBP were right-skewed and natural log transformed to fit normality assumptions in statistical models.

We have adjusted for specific gravity and urine dilution as time-varying covariates in our regression models to be consistent with our previously published studies. One can adopt alternative approaches to standardize the urinary phthalate measures, for example, by regressing phthalate levels on these covariates and using the resultant residuals as exposure in the subsequent outcome-exposure model. We conducted a sensitivity analysis to compare our simultaneous adjustment strategy to this two-step strategy and noted that there is no systematic pattern in terms of enhanced significance in one adjustment strategy versus another (data not shown). Thus, for all of the methods presented we opted to include specific gravity as a covariate in the statistical models we fit.

The primary dilemma for examining exposure data with this time-varying structure is how to account for the longitudinal features of the exposure trajectory in a final disease risk model with the binary outcome, conditional on the complete set of exposure measures. This type of problem is somewhat unique, as in most regression settings the independent variable and the dependent variable are either both cross-sectional, both longitudinal, or the outcome is longitudinal with a single baseline measure of exposure. A commonly used approach in the realm of studying environmental exposures in relation to preterm birth is to examine multiple cross-sectional models, e.g., the relationship between trimester specific exposure levels in association with preterm birth [8, 9, 11, 12]. This approach enhances the burden of multiple hypothesis testing. Another previously employed solution is to include the exposure measures at each time point simultaneously in a multivariate logistic regression model [12], however this too is problematic as these measures are likely to be correlated, leading to inflated standard error estimates and erroneous odds ratio estimates. Additionally, studies with this type of data structure commonly model an average of repeated exposure measures, but doing so wastes all information on temporal variation of the exposure and reduces exposure variability.

In each section of the results, we will examine these as well as six additional methods to elucidate possible modeling strategies that might be useful in different applications. For each method we examine the association between a single binary variable (preterm birth status) and a continuous variable with repeated biomarker measurements (MEHP or MBP) controlling for time-invariant (maternal age at visit 1, race/ethnicity, health insurance provider, education level, and pre-pregnancy body mass index [BMI]) as well as time-varying (urinary specific gravity as well as time of day of urine sample collection) covariates. In previous analyses published by this group, different combinations of covariates were included based on their improvement of specific model types for each phthalate metabolite. For consistency and illustration purposes in this paper, all of the above covariates were included for each model presented and this may account for incongruence between these and other previously published results.

For notational convention, we will let *Y*_*i*_ denote the binary outcome for subject *i*, *X*_*ij*_ denote the continuous measurement corresponding to subject *i* at visit *j* (occurring at time *t*_*ij*_, with time measured in units of gestational days), and *Z*_*ij*_ denote the vector of covariates where *i* = 1, …, *N* and *j* = 1, …, *n*. For convenience, we also let *X*_*i*_ = (*X*_*i*1_, *X*_*i*2_, …, *X*_*in*_)^*T*^ and  denote all exposure and covariate data available for subject *i.* Each subject may not have all *n* measurements and in that case we will let *n*_*i*_ denote the number of exposure measures available per subject and use *j* = 1, …, *n*_*i*_. R codes for each method are included in Additional file 1 and are available at http://www-personal.umich.edu/~yinhsiuc/Rcode-longitudinal.html.

## Results and discussion

### Standard methods

#### Multiple logistic regression model

A simple way of modeling this association is to regress *Y*_*i*_ on all available *X*_*ij*_ s (for *j* = 1, …, *n*) controlling for the mean vector of covariates , that is:


Note that, since this model requires a complete set of predictors *X*_*ij*_, it will use only the subjects having all exposure data at all *n* visits, a major limitation of such an approach. Using our dataset, we modeled urinary phthalate metabolite levels measured at four visits in one model predicting preterm birth. MEHP at visit 3 only was significantly associated with increased odds of preterm birth (Table 1). Likewise, MBP at visit 3 was suggestively associated with increased odds of preterm delivery but levels measured at other visits showed no association (Table 1).

**Table 1:** **Odds of preterm birth from multiple logistic regression models (method** **multiple logistic regression model** **)**

	MEHP	MBP
	Odds ratio (95% confidence interval)	Odds ratio (95% confidence interval)
Visit 1	1.08 (0.85, 1.38)	0.84 (0.56, 1.25)
Visit 2	0.93 (0.70, 1.24)	1.17 (0.83, 1.66)
Visit 3	1.33 (0.99, 1.79)	1.49 (0.98, 2.27)
Visit 4	1.11 (0.83, 1.48)	1.17 (0.80, 1.72)

N = 282 for MEHP and MBP models. Odds ratios in association with ln-unit increase in urinary phthalate metabolite concentration at each study visit. Models adjusted for maternal age at visit 1, race/ethnicity, health insurance provider, education level, BMI at visit 1, and urinary specific gravity and time of day of sample collection at each study visit.

There are several potential problems with this analysis. First, collinearity between the repeated of measures of phthalate metabolites may be an issue because it may lead to inflated standard errors and plausibly change the direction of estimates of *β*_*j*_ s [13]. We examined the correlation matrix for both the phthalates by study visit and found relatively high collinearity between visits, indicated by pairwise correlation coefficients ranging from 0.26 to 0.48 for MEHP and from 0.50 to 0.57 for MBP (Table 2) [11]. The inverse associations between preterm birth and MEHP at visit 2 and MBP at visit 1 might indicate unstable estimation resulting from this collinearity between the longitudinal phthalate measures.

**Table 2:** **Pairwise correlation coefficients for MEHP (upper triangle) and MBP (lower triangle)**

	Visit 1	Visit 2	Visit 3	Visit 4
Visit 1		0.33	0.26	0.26
Visit 2	0.52		0.48	0.37
Visit 3	0.57	0.55		0.41
Visit 4	0.53	0.49	0.56	

A second issue with this method is interpretation of results, as each regression coefficient represents how the phthalate level at a certain visit is associated with preterm delivery status after controlling for the measures at other visits, and it is unrealistic to vary only one of a series of longitudinal measures with other measures fixed. Finally, as mentioned before, this approach requires that *X* be measured at, at least nearly, a uniform set of time points in order to make *β*_*j*_ s interpretable, as the *X* s are indexed by visit and not continuous time. If the missing exposure observations are infrequent and the data are almost complete and missing completely at random, approximating an unbalanced data set by a balanced one is subject to limited loss of efficiency. Otherwise, the efficiency loss might be considerable and the approximation may result in serious bias [14]. In this case, as many of the preterm cases had already delivered by visit 4 and there was consequently potential missingness explained by the outcome (missing at random) in phthalate measurements at this time point, the results from this method may be biased.

We also adapted a Bayesian method originally proposed by Warren et al. [15] for spatio-temporal data to handle longitudinal exposure measurements in association with a binary outcome of interest. The Bayesian method includes exposure at each visit (1 to *n*) simultaneously in the outcome model (e.g., multiple logistic regression) but introduces a specificform of covariance structure for the Gaussian prior on the coefficients . The *a priori* structure handles the correlation among the effects of exposures at each visit through shrinkage of the regression coefficients, just like Ridge regression, by assuming that correlation between temporally and spatially proximate coefficients is higher. However, the advantage of this method may be quite limited for studies utilizing a small number of repeated maternal biomarker measurements during pregnancy, and may be more practical for applications where a large number of exposure measurements, such as ambient air monitoring data. In our application the Bayesian approach yields results very similar to those from the multiple logistic regression model with slight attenuation towards the null. However, the Bayesian method handles correlated within-subject exposures across pregnancy in a more principled manner.

This method and the other three standard methods introduced later in this section assume a linear relationship between exposure and response. Adding higher-order polynomial term(s) or replacing the linear term with a smoothing spline term in the framework of Generalized Additive Models (GAM) can accommodate non-linearity in any of these methods.

#### Parallel cross-sectional logistic regression models

A commonly-used approach to circumvent the collinearity problem from Method Multiple Logistic Regression Model is to fit *n* separate cross-sectional models for each visit as:


In the example data, MEHP and MBP measures at each visit are positively correlated with preterm delivery although none of the effect estimates are statistically significant (Table 3). Odds ratios for preterm delivery range from 1.10 to 1.17 and from 1.15 to 1.34 with an ln-unit increase in MEHP and MBP, respectively, after adjustment for covariates. One major drawback of this method is that there is no straightforward way to combine the results from multiple regression models and assess the aggregate effect of *X* on *Y*. Also, if desired, controlling for family-wise error rate (e.g., using Bonferroni correction) may be conservative because of the varying degree of dependency between the multiple tests. If instead of fitting separate models we jointly estimate *β*_*j*_ s using the generalized estimating equations (GEE) approach described by Sanchez et al. [6], it is possible to circumvent some of these concerns as well as test the differences in associations across visits (i.e., *H*_0_ : *β*_1_ = *β*_2_ = … = *β*_*n*_). In our example, there are no significant differences in the associations across four study visits for either MEHP (p = 0.95) or MBP (p = 0.81) based on this GEE-based joint estimation method. The odds ratios for preterm delivery based on joint estimation are identical to those in Table 3 with slightly narrower confidence intervals for MEHP and slightly wider confidence intervals for MBP (data not shown).

**Table 3:** **Odds of preterm birth from parallel cross-sectional logistic regression models (Method** **Parallel cross-sectional logistic regression models** **)**

		MEHP	MBP
	N	Odds ratio 95% confidence interval	Odds ratio 95% confidence interval
Visit 1	456	1.10 (0.93, 1.30)	1.19 (0.95, 1.48)
Visit 2	407	1.12 (0.93, 1.35)	1.15 (0.91, 1.46)
Visit 3	392	1.17 (0.96, 1.43)	1.23 (0.97, 1.55)
Visit 4	322	1.11 (0.86, 1.43)	1.34 (0.98, 1.83)

Odds ratios in association with ln-unit increase in urinary phthalate metabolite concentration at each study visit. Models adjusted for maternal age at visit 1, race/ethnicity, health insurance provider, education level, BMI at visit 1, and urinary specific gravity and time of day of sample collection at each study visit.

#### Model using mean exposure across visits as a summary

The third method regresses the binary variable on the subject-specific averages of the continuous time-varying exposure variable in a logistic regression framework as follows:


where . This approach is the first attempt to summarize the longitudinal information into one measure and is useful when there is no particular trend in *X* or the trends over time are similar for subjects with either binary outcome (*Y*_*i*_ = 1 or *Y*_*i*_ = 0, preterm or term). If this is true,  adequately captures the longitudinal feature of *X* to differentiate the two groups. Since phthalate measures are log-normally distributed, subject-specific geometric rather than arithmetic averages were considered as summary predictors [10]. This method has been utilized previously to examine associations within this dataset but in that analysis visit 4 measures were excluded because of the aforementioned bias in availability of measures at that time point [10]. In the present analysis all available measurements were included in subject-specific averages, and averages were ln-transformed for modeling purposes. The time-varying covariates were also averaged to create subject specific average covariate values ().

The association between average MEHP levels and preterm birth was statistically significant (β = 0.27, standard error [SE] = 0.13, adjusted odds ratio [aOR] = 1.30, p = 0.05, N = 417). MBP average was also suggestively associated with preterm birth (β = 0.24, SE = 0.14, aOR = 1.28, p = 0.08, N = 417). An advantage of this method is that the interpretation of odds ratios is more natural. Odds of preterm delivery was 1.30 times and 1.28 times higher for mothers with an ln-unit increase in average urinary MEHP or MBP concentration over the course of pregnancy, respectively.

One difficulty in this method is the treatment of time-varying covariates. For example, to control for time of day of urine sample collection (before vs. after 1 pm), we also used an average of time of day at each study visit. However, the averaged variable is difficult to interpret and may not accurately reflect differences in urinary phthalate metabolite concentrations by time of day. This method may be additionally limited if the data are unbalanced and not missing at random, as mentioned above, or if there are trends in biomarker measures over time that are more relevant to the outcome than the biomarker levels themselves.

#### Model using maximum exposure value across visits as summary

This method resembles the Model using mean exposure across visits as a summary, except now we regress the binary variable on the maximum, rather than the average, of the continuous variable:


where  and  is defined as the vector of covariates at the visit of which the continuous variable assumes its maximum value for subject *i*. In other words, time-varying covariates corresponding to the maximum value of *X*_*i*_ are included in the final model. This approach may be more appropriate when the association between *Y* and *X* is not driven by the longitudinal trend of *X* or an average level but rather an acute or extreme instance of exposure.

Associations using this method were not statistically significant for MEHP (β = -0.07, SE = 0.09, aOR = 0.94, p = 0.46, N = 442) or MBP (β = 0.10, SE = 0.12, aOR = 1.11, p = 0.39, N = 444). This method may be inappropriate for examining associations with phthalates, which are metabolized and excreted quickly but may be measured at very high levels following a recent exposure.

### Two stage methods

#### Two stage mixed effects model

A two-stage approach can relax the assumption for the Model using mean exposure across visits as a summary or the Model using maximum exposure value across visits as summary, that the longitudinal trend is irrelevant in terms of detecting the association between *X* and *Y*. In this approach, the longitudinally time-varying exposure is first modeled as a function of time (e.g., using random slopes and intercepts) and at the second step best unbiased linear predictor (BLUP) estimates of these random coefficients are used as predictors in a logistic regression model. The formulation for this model is as follows:


where *a*_0*i*_ and *a*_1*i*_ are the random intercept and random slope jointly distributed as bivariate normal random variables representing the longitudinal trend of *X*, *α*_0_ and *α*_1_ are corresponding fixed effects, and *ϵ*_*ij*_ is the error term distributed independently of the random effects. In this method the subject-specific time trends of *X* from Stage 1 are extracted and modeled in the second stage along with relevant covariates. This method is appropriate if it seems plausible that the predicted subject-specific intercepts and slopes for *X* provide an accurate summary characterization of distinct patterns across the two outcome groups. If the exposure variable *X* is not normally distributed, the joint normality assumption for the random intercept and random slope may be inappropriate. As mentioned in the Methods section, we natural-log transformed MEHP and MBP prior to analysis in order to better approximate the normality assumption. If needed, more sophisticated methods for treating specific exposure distributions have been suggested by Arellano-Valle et al. (skew-normal linear mixed model) [16] and Zhang et al. (log-gamma linear mixed model) [17].

Subject-specific slopes and/or intercepts from Stage 1 can be simultaneously included as continuous predictors in the Stage 2 logistic regression model. Alternatively, one can cluster fitted intercepts and slopes from Stage 1 on a 2-dimensional Euclidean space and model the resulting clustering index (*â*_0*i*_, *â*_1*i*_ ) in the Stage 2 logistic regression model. This second method effectively groups subjects based on similarities in trends in exposure levels over pregnancy and may have greater power over modeling BLUP estimates. Additionally, if desired, this method can flexibly accommodate a non-linear trend in Stage 1 with higher-order terms, such as quadratics (e.g., *â*_2*i*_ or *α*_2_) or curvature characteristics (e.g., 2(*â*_2_ + *â*_2*i*_) + (*â*_1_ + *â*_1*i*_)) as predictors of the binary outcome in Stage 2. Finally, if the relationship between exposure and response is found to be non-linear, one can include a smooth functional representation of the summarized characteristics from Stage 1 in Stage 2 to account for the non-linearity.

In the example analysis, we modeled subject-specific slopes and intercepts extracted from Stage 1 continuously in Stage 2. Using this method, MEHP exhibits evidence suggestive association (*β*_1_=0.35, SE = 0.20, aOR = 1.43, p = 0.08, N = 417) between the subject-specific predicted intercepts and preterm delivery, but the effect estimate for predicted slope was not statistically significant. This result suggests that the mean MEHP exposure level, as indicated by the subject-specific predicted intercepts, was associated with preterm birth, but trends in levels across pregnancy were not contributors to the outcome. With respect to MBP, the predicted slopes and predicted intercepts were highly correlated in the Stage 1 model suggesting that the additional inclusion of random slope for gestational age at sample collection was not necessary. We thus refit the model without random slope and used the random intercept only as a predictor in the Stage 2 model. An ln-unit increase in the predicted intercept for MBP was associated with an increased odds of preterm birth (*β*_1_ =0.32, SE = 0.17, aOR = 1.37, p = 0.07, N = 417), which is again similar to the interpretation from the mean model.

**Figure 1:**
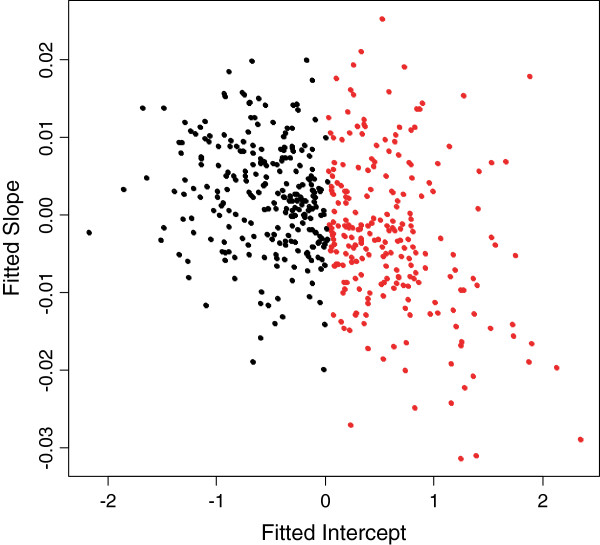
**Scatterplot of fitted intercepts and slopes from the mixed effects model with MEHP regressed on gestational age.**

We also examined the effect of dividing subjects into two clusters based on a k-means clustering with subject-specific predicted intercepts and slopes plotted on the two-dimensional space described above. This was performed only for MEHP, as subject-specific slopes were not important for predicting MBP concentrations. For MEHP, the clearest separation occurred based on the predicted intercept, and the predicted slope term was less important (Figure 1). In the Stage 2 model the clustering index for MEHP was marginally associated with preterm delivery (*β*_1_ =0.43, SE = 0.26, aOR = 1.54, p = 0.09, N = 417). The individuals classified in the “greater predicted intercept” group (red dots) had 1.54 times the odds of having a preterm delivery compared to the “smaller predicted intercept” group (black dots).

A major drawback of this model is that BLUP estimates or clustering uncertainty from the Stage 1 model is not accounted for in the Stage 2 analysis, which may lead to biased results. A Bayesian analysis or an analysis based on a joint likelihood of the longitudinal model and the final logistic model (as done in [7]) can ensure proper propagation of uncertainty. The functional methods discussed in Methods based on clustering section involve joint estimation, but suffer from having sparse measurements over time.

#### Generalized additive mixed model to contrast exposure trajectories

In all of the previous methods, we treated *X* as the independent variable and *Y* as the dependent variable in the regression framework. However, if the focus is not to establish causality, or characterize risk of preterm birth, a reverse temporal model that treats *X* as a longitudinal dependent variable and *Y* as a time-invariant independent binary variable can be used to contrast exposure trajectories in the two groups. To that end, we use the following generalized additive mixed model (GAMM):


where *b*_0*i*_ is the random intercept and *f*_1_(.) and *f*_2_(.) are smooth functions, represented by a natural spline in this example. Time is equivalent to gestational age at urine sample collection. This model naturally accounts for the longitudinal nature of *X* and the trend curves can be depicted parametrically or non-parametrically. While the model is not interpretable in terms of temporality, since the occurrence of *X* precedes *Y*, the results may provide information about the differences in *X* for each level of *Y* over the time course of pregnancy.

**Figure 2:**
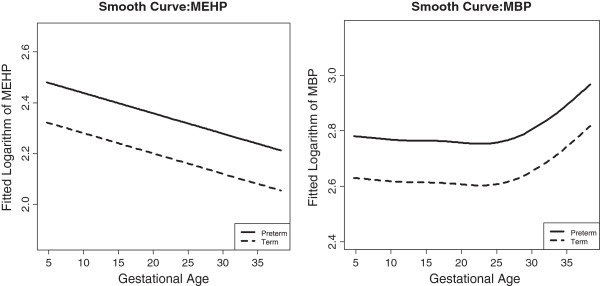
**Fitted smooth curves between phthalate levels and gestational age.**

The fully flexible saturated model allows a separate smooth curve for each level of *Y*, which is equivalent to allowing an unconstrained interaction term between *Y* and gestational age. In the present analysis we started with these freely fitted smooth curves (MEHP or MBP predicted by gestational age at sample collection) in mothers who delivered preterm compared to mothers who delivered term. This has been illustrated previously in this dataset [11] and is replicated in Figure 2. The estimated degrees of freedom (EDF) for the difference between the two curves was 2 for both MEHP and MBP models, indicating a linear difference in the two groups across gestation. Additionally, the slope of the linear difference in the two groups was not significantly different from zero (type one error rate = 0.05), further confirming a constant difference in MEHP or MBP levels in the two groups. There was a nearly significant interaction between gestational age at sample collection and preterm birth for both MEHP (p = 0.09) and MBP (p = 0.07), which parallels the significant differences in cases and controls observed in previous methods. However, to fully characterize differences using the exposure trajectories from this method a larger sample size and/or additional study visits would be necessary.

This method accounts for potential non-linearity in the relationship between exposure and gestational age. However, there is no clear way to examine the non-linear relationship between exposure and preterm birth status in this model. A possible alternative is to regress preterm birth status on a tensor product smoother of gestational age and exposure [18]. However, the difficulty of interpreting these results and the sparseness of our data limit the applicability of this alternative in this situation.

### Methods based on clustering

#### Gaussian mixture model by clustering the exposure values

This method treats the longitudinal measures in *X*_*i*_ = (*X*_*i*1_, *X*_*i*2_,…, *X*_*in*_)^*T*^ as a vector and distinguishes the subjects with *Y*_*i*_ = 1 vs. *Y*_*i*_ = 0 by characterizing the *X*_*i*_ vectors of each group. If the dataset is almost balanced in a sense that every subject has the same number of observations for *X* with minimal amount of missingness, we can assume that each *X*_*i*_ follows a multivariate normal distribution with an unknown mean and variance. In this method, we hypothesize that there are *K* latent multivariate normal distributions with different means and variances from which *X*_*i*_ s are drawn [19]. Subjects with *X*_*i*_ s from some of the latent distributions will have a higher probability of being *Y*_*i*_ = 1 compared to subjects with other latent distributions for *X*_*i*_*.* A two-stage procedure can then be devised to identify clusters of *X*_*i*_ that have an increased probability of *Y*_*i*_ = 1. In the first stage, subject-specific exposure vectors, *X*_*i*_ s, are clustered based on Gaussian distributions with a predetermined number of clusters (*K*). Each subject is grouped into one of the *K* clusters. In the second stage, we then regress *Y*_*i*_ on this clustering index along with the covariates as:


where *C*_*i*_ is the clustering index, which may be more than one-dimensional when *K* > 2. A limitation to this method is that *X* has to be balanced (*n*_*i*_ = *n*). Thus, in the present analysis, the dataset had to be restricted to subjects with phthalate measurements available at all four study visits (N = 280), which may bias results as many fewer cases had observations available at visit 4 (median gestational age = 35 weeks). Nevertheless, we performed the analysis to illustrate the application of this method. An additional limitation of this method is that *n* must be relatively small compared to the number of subjects, *N*. If *n* is too large, there may be more parameters in the model than there are subjects, and the covariance matrices would have to be restricted [19]. In this dataset *n* (4) was sufficiently smaller than *N* (280). As with other applications we natural-log transformed MEHP and MBP to better approximate the normality assumptions required for this method. As an alternative, non-Gaussian clustering methods may be performed as suggested by Banfield and Raftery [20].

**Figure 3:**
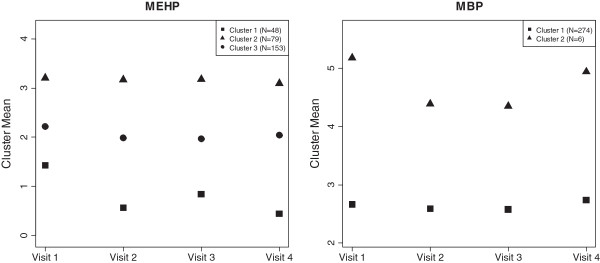
Estimated mean of clusters* suggested by the Gaussian mixture model, stratified by study visit.

We selected the optimal number of clusters according to Bayesian information criterion (BIC) approximation for model-based clustering [21]. The number of clusters chosen for MEHP and MBP were three and two, respectively. The means of the phthalate metabolite concentrations within each cluster by study visit are displayed in Figure 3. In Stage 2 we modeled each clustering index in relation to odds of having a preterm delivery. For MEHP, the odds of having a preterm birth for subjects in cluster 2 was 1.84 times (*β*_1_ =0.61, SE = 0.65, p-value = 0.35) the odds for subjects in cluster 1; subjects in cluster 3 had 2.09 times (*β*_2_ =0.74, SE = 0.55, p-value = 0.18) the odds of having a preterm birth compared to subjects in cluster 1. For MBP, subjects in cluster 2 had 5.44 times the odds of having a preterm birth (*β*_1_ =1.69, SE = 0.87, p = 0.05) compared to subjects in cluster 1. However, for MBP, 274 subjects fall into cluster 1 and only 6 fall into cluster 2, making these results unreliable. The imprecision of the MEHP estimates and the instability in the Stage 1 clustering for MBP may stem from the small sample size of the complete dataset. Additionally, as suggested by the results from the method using Generalized additive mixed model to contrast exposure trajectories, the trajectories characterized by vectors in this method may not be meaningful for phthalates or identifiable with only 4 exposure measurements.

#### Functional clustering model

Whereas the previous method entailed clustering based on Gaussian distributions of *X*, here we employ k-means clustering based on non-parametric distributions. This method utilizes principal points which summarize important features of the relationship between *X*_*ij*_ and *t*_*ij*_, or, in this example, the relationship between urinary phthalate metabolite concentration and gestational age at sample collection [22, 23]. The principal points, which can be based on either raw curves or derivatives, effectively reduce the functional data to multivariate data, and enable subsequent k-means clustering. The advantage of functional clustering compared to Gaussian mixture clustering is that it does not require *X* to be balanced. Thus, this method is preferable for the present example compared to Method (Gaussian mixture model by clustering the exposure values).

This two-stage procedure can be conducted in two ways. The first is to cluster the curves of *X*_*ij*_ against *t*_*ij*_ first, and then to regress the binary outcome *Y*_*i*_ on the clustering index along with covariates in the second stage. A drawback to this method is that clustering of the curves may be dependent on one of the covariates, e.g., the race/ethnicity of the subject. An alternative approach is to first regress *X*_*ij*_ on all covariates of interest, then to cluster curves of the residuals vs. *t*_*ij*_ and use those clusters in a logistic regression model. For either variation of this method, the dependency between *Y*_*i*_ and the clustering index can be tested with a chi-square test (with degrees of freedom = *K* - 1).

**Figure 4:**
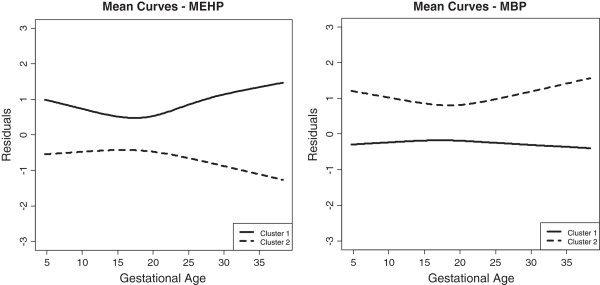
Mean trajectories of clusters* based on functional curves of phthalates vs. gestational age. *Clusters are constructed based on functional k-means clustering (Functional clustering model) of the smooth curve of residuals (from a regression model of MEHP or MBP on relevant covariates) against gestational age. N=443 for each phthalate model.

In the present dataset we fixed the number of clusters to two for this method. In order to determine whether the functional clustering was dependent on covariate distributions only, rather than phthalate exposure, we conducted two-sample t-tests and chi-square tests for the continuous and categorical variables, respectively, with grouping based on the clustering results (data not shown). For MEHP, clustering was strongly associated with maternal race/ethnicity, BMI, and urinary specific gravity. For MBP, most covariates were significantly associated with the clustering index. Thus, in this example, functional clustering based on a model of *t*_*ij*_ vs. the residuals from the model of *X*_*ij*_ regressed on all covariates was more appropriate. Figure 4 displays the mean smooth curves of phthalate metabolite residuals vs. gestational age at sample collection for the two clusters identified. Subjects in cluster 1 had an elevated odds of having a preterm birth compared to subjects in cluster 2 for both MEHP (aOR = 1.60, p = 0.03) and MBP (aOR = 1.27, p = 0.36).

#### Functional logistic regression model

In this method, we assume that the binary outcome depends on the longitudinal predictor variable along with the covariates of interest through the functional logistic regression model proposed by Muller et al. [24] as follows:


where *β*(*t*) is the time-varying coefficient for the longitudinal predictor variable. The interpretation is that the log odds of *Y*_*i*_ = 1 would increase by amount  from time point *t*_1_ to time point *t*_2_ with a unit increase in the predictor *X*_*i*_(*t*) across the time interval *t*_1_ to *t*_2_. In the context of this example, the odds of being born preterm would be dependent on aggregate phthalate concentrations across gestation periods (e.g., from Visit 1 to Visit 4).

This method utilizes functional principal component (FPC) scores to summarize the important features of functional curves of exposure levels over time and include the scores in the regression model. The FPC scores can be estimated via the conditional expectation method proposed by Yao et al. [25]. The number of basis functions used to construct the functional curves and the number of leading FPC scores included (*L*) can be determined by the leave-one-curve-out cross-validation score suggested by Peng and Paul [26]. A global test of *H*_*o*_ : *β*_1_ = *β*_2_ = … = *β*_*L*_ = 0 such as *L* -degree-of-freedom likelihood ratio test (LRT) can be conducted to investigate the global association between longitudinal *X* and the stationary binary outcome *Y*_*i*_.

The number of basis functions and the number of leading FPC scores selected by modified BIC are nine and two, respectively, for both MEHP and MBP. The 2-df LRT statistic for testing if there is an overall association between MEHP and preterm delivery was 3.48 (p = 0.18). For MBP, the 2-df LRT statistic was 4.81 (p = 0.09). The inclusion of the estimated FPC scores does not significantly improve the proportion of variance explained by the model for either phthalate metabolite. In other words, there is not sufficiently strong evidence for an association between MEHP or MBP measures summarized by FPC analysis and preterm delivery. This may mean that: a) the changes in phthalate levels over pregnancy are not associated with preterm birth; or b) that there is not sufficient data (sample size or number of observations) to detect associations with phthalate change over pregnancy and preterm birth in this dataset. For other exposures or biomarkers that have more substantial increases or decreases across gestation or for a study with larger sample size and/or additional repeated measures this method may provide more useful information.

The clustering strategies underlying each method in the Methods based on clustering section, based on the longitudinal measures of the exposure in the original scale, assumes a linear relationship, albeit indirectly, between exposure and response. If desired, one can perform clustering based on the transformed exposure levels, such as *X*^2^ and *e*^*X*^, in order to account for relationships that are non-linear in nature.

## Conclusions

In the context of examining repeated maternal biomarkers during pregnancy in association with preterm birth, we described 9 statistical methods that may be useful for fully utilizing longitudinal characteristics of data in future studies. Each has strengths and limitations and may be suitable in some circumstances but not others based on both characteristics of the predictor as well as goal of the analysis (summarized in Table 4). For identifying windows of vulnerability, examining information from each study visit simultaneous or separately (Multiple logistic regression model and Parallel cross-sectional logistic regression models) or modeling individual (Two stage mixed effects model) or population level (Generalized additive mixed model to contrast exposure trajectories) patterns of exposure in relation to the health outcome may be most useful. Sanchez et al. previously contrasted utility of these approaches for identifying particularly important windows of exposure [6]. This case-study alternatively presents methods for leveraging multiple exposure measurements over time to more powerfully detect a true association with a non-time-varying outcome. Such a relationship may arise from: 1) Exposure during a particularly sensitive window of pregnancy; 2) Generally elevated levels of exposure across gestation; 3) An acute exposure; or 4) A change in exposure over time, e.g., an increasing trend over the course of weeks or months.

**Table 4:** **Advantages and limitations to methods for modeling repeated biomarkers of exposure in association with a binary, non-time-varying outcome**

	Advantages	Limitations
**Method**	- Simple implementation	- Collinearity in longitudinal phthalate measures can cause instable effect estimates and inflated variance estimates
Multiple logistic regression model	- Jointly account for longitudinal phthalate measures in one model	- Requires time points to be uniform
		- Only the subjects with complete data are used
		- Difficult interpretation
**Method**	- Simple implementation	- No straightforward way to combine results from multiple regression models to assess aggregate effect of phthalate levels on preterm birth
Parallel cross-sectional logistic regression models	- Subjects with incomplete data can be retained	- Control for family-wise error rate using Bonferroni correction may be too conservative
	- Simple interpretation	
**Method**	- Simple implementation	- Difficult to handle time-varying covariates
Model using mean exposure across visits as a summary	- Simple way to account for and summarize longitudinal phthalate measures	- Limited if data are unbalanced and/or not missing at random
	- Straightforward interpretation	- Trends of phthalate measures relevant to the outcome may be missed
	- Improved power when exposure has poor stability over time and exposure levels themselves are most relevant to the outcome	
**Method**	- Simple implementation	- May be inappropriate when maximum concentrations are indicative of recent rather than acute exposure
Model Using maximum exposure value across visits as summary	- Straightforward interpretation	- Deposition of time-varying covariates is questionable
	- Powerful when the association is not driven by the longitudinal trend and/or average level but rather an acute instance of phthalate exposure	
**Method**	- Flexible modeling of exposure pattern over time in Stage 1	
Two stage mixed effects model	- Examines effect of characteristics carried from Stage 1 in Stage 2	- Uncertainty from Stage 1 is not incorporated in Stage 2 which may lead to biased results
	- Naturally accounts for between subject heterogeneity	- May not be useful when phthalate levels are unstable over time
**Method**	- Accounts for longitudinal nature of exposure	- Not temporally logical
Generalized additive mixed model to contrast exposure trajectories	- Trends of exposure can be depicted parametrically or non-parametrically for each group	- Risk cannot be estimated
**Method**	- Allows risk estimation based on cluster identity	- Requires dataset to be balanced and complete
Gaussian mixture model by clustering the exposure values	- Characteristics of each cluster well-depicted by a multivariate Gaussian distribution	- Requires longitudinal phthalate measures to follow a multivariate Gaussian distribution
	- Direct interpretation	- Subtle characteristics cannot be captured by the first two moments
		- Computationally expensive
**Method**	- Accounts for longitudinal nature of the exposure and time-varying covariates	- May be underpowered if trends of phthalate levels are unimportant
Functional clustering model	- Allows risk estimation based on cluster identity	- Trends are unreliable if the data are sparse with (few time points for each subject)
	- Does not require exposure to be balance and complete	
	- Direct interpretation	
**Method**	- Accounts for longitudinal nature of the exposure and time-varying covariates	- Difficult interpretation
Functional logistic regression model	- Does not require exposure to be balanced and complete	- Trends are unreliable if the data are sparse (few time points for each subject)
	- Longitudinal information is entirely retained in FPC scores	- Choice of number of principal and number of basis function via BIC is ad-hoc

The preponderance of the research on environmental exposures during pregnancy and preterm birth examine an association with generally elevated levels of exposure, and utilize one metric during gestation. The Model using mean exposure across visits as a summary and random intercepts from the Two stage mixed effects model use repeated measures that can more powerfully detect such an effect than any single measurement model, e.g. the Multiple logistic regression model and the Parallel cross-sectional logistic regression models, regardless of the number of exposure measurements available. Notably, employing the two stage model from the Two stage mixed effects method did not improve results obtained from simply taking an average of exposures in this example, although the Two stage mixed effects model accounts for time-varying covariates in a more sensible manner.

Fewer studies have expressed interest in identifying whether an acute exposure at any time point or during sensitive period is associated with preterm birth. To some extent the methods for investigating windows of vulnerability address this question, as do some studies utilizing ambient air pollution measures and survival analyses [27, 28]. Additionally, the Maximum Model (Model using maximum exposure value across visits as summary) may serve to this end, and the functional logistic regression approach (Functional logistic regression model) has been used recently to identify windows of susceptibility for long term trajectories of exposure with rich repeated measurements, and for studying how genetic factors may modify these windows [29]. These approaches may not be useful for urinary phthalate metabolites, or other biomarkers that are highly variable over time, as high concentrations may be indicative of temporally recent rather than acute exposure.

The contribution of temporal changes in environmental exposures to preterm birth is relatively understudied, particularly in research utilizing biomarkers of exposure measurement during pregnancy. However, it is plausible that these patterns may contribute to prematurity and other adverse birth outcomes when steady exposures do not. For air-pollution studies or ambient monitoring data measured at a finer time scale where personalized measures are not needed, this temporal feature can and has been studied in greater detail, for example, as presented in Warren et al. [15]. The Two stage mixed effects model, when incorporating random slopes, and the Gaussian mixture model by clustering the exposure values, Functional clustering model, and Functional logistic regression model, are different ways of capturing the additional information that repeated measures over time provide that may contribute more than pure exposure measure contributions to preterm birth. Additionally, though not capable of quantifying an effect, the Generalized additive mixed model to contrast exposure trajectories can characterize patterns and establish differences in preterm vs. term groups. These methods were not useful for further understanding the relationship between phthalate exposure and preterm birth, potentially due to few exposure measurements, the instability of urine levels, or because phthalates are not chemicals whose patterns over pregnancy contribute to this outcome. These methods may be more useful for datasets with higher frequency of exposure assessment, with a more temporally resolved structure. Collection of a dense set of measurements may not be practically feasible for studies analyzing expensive biomarkers during a short time window (40 weeks gestation) in a large prospective cohort study. However, they may be applicable in studies utilizing less costly exposure metrics, such as ambient air monitoring or using simpler, less expensive and non-invasive techniques for exposure assessment. In addition to requiring this robust data structure, the temporal methods have other limitations. The Two stage mixed effects model, Gaussian mixture model by clustering the exposure values, and Functional clustering model ignore uncertainty in the first step estimation or clustering and thus underestimate the standard error in the final odds ratio, increasing the likelihood of a false positive in the results. The Functional logistic regression model is more desirable because it does not require these two steps and carries out inference based on a joint likelihood. Another limitation is that these methods do not appropriately account for drop-out which is inherent in a longitudinal study. In this case-study, missingness at visit 4 is likely related to the outcome of interest, preterm birth, leading to a missing at random mechanism [30]. In addition, missingness could also be related to other unmeasured covariates. We recommend that sensitivity analyses with respect to the parameters of the drop-out probability model be performed whenever this may be the case. If a probability model for drop-out can be validly constructed, one can leverage an inverse probability weighting approach. Despite these limitations, the temporal methods described may provide new insight into the study of environmental exposures and prematurity.

In conclusion, the methods exemplified in this case study may be of great use in future epidemiologic research projects intended to: 1) Elucidate the complex relationships between environmental chemical exposures and preterm birth; 2) Investigate biological mechanisms in prematurity using repeated measures of maternal factors throughout pregnancy; and 3) More generally, address the relationship between a longitudinal predictor and a binary, non-time-varying outcome.

## Electronic supplementary material

Additional file 1: **R Code for Statistical Methods.** (DOCX 29 KB)

## Abbreviations

MEHP: Mono-2-ethylhexyl phthalate
MBP: Mono-*n*-butyl phthalate
ICC: Intraclass correlation coefficient
CI: Confidence interval
BMI: Body mass index
GEE: Generalized estimating equations
SE: Standard error
aOR: Adjusted odds ratio
BLUP: Best linear unbiased predictor
GAMM: Generalized additive mixed model
EDF: Estimated degrees of freedom
BIC: Bayesian information criterion
FPC: Functional principal component
LRT: Likelihood ratio test.
