# Supplementary material for: Statistical methods for modeling repeated measures of maternal environmental exposure biomarkers during pregnancy in association with preterm birth
Source: Environ Health. 2015 Jan 26;14:9. doi: 10.1186/1476-069X-14-9 (PMC4417225; doi:10.1186/1476-069X-14-9)
Supplement: Supplementary file 1 — Additional file 1: R Code for Statistical Methods. (DOCX 29 KB) [file 12940_2014_845_MOESM1_ESM.docx]

**Additional file 1: R Code for Statistical Methods**

dat1.1: a matrix containing exposure variables, response variables, and covariates in long format

column 1: Preterm birth status (binary)

column 2: Maternal age at visit 1 (continuous)

column 3: Subject ID

column 4: Race / Ethnicity (categorical)

column 5: Health insurance provider (categorical)

column 6: Education level (categorical)

column 7: Pre-pregnancy body mass index (categorical)

column 8: Gestational age (continuous)

column 9: MEHP measurements (continuous)

column 10: MBP measurements (continuous)

column 11: Urinary specific gravity (continuous)

column 12: Time of day of urine sample collection (binary)

column 13: Which number of visit

*## Method 3.1.1: Multiple Logistic Regression Model*

*for(i in 1:2) {*

*temp.dat1 <- reshape(data.frame(dat1.1[, c(1:8, i+8, 11:13)]),*

*timevar = "visit", idvar = "id", direction = "wide")*

*sgavg <- apply(temp.dat1[, c(10, 20, 30, 40)], 1, function(x) mean(x, na.rm = TRUE))*

*temp.dat1$bmi_cat_new.1 <- as.factor(temp.dat1$bmi_cat_new.1)*

*temp.dat1$race_cat_new.1 <- as.factor(temp.dat1$race_cat_new.1)*

*temp.dat1$edu_cat_new.1 <- as.factor(temp.dat1$edu_cat_new.1)*

*temp.dat2 <- cbind(temp.dat1[, 2:7], sgavg, temp.dat1[, c(41, 9, 19, 29, 39)])*

*mod.temp <- glm(PRETERM.1 ~., data = temp.dat2, family = binomial)*

*mod.fourcov[[i]] <- mod.temp*

*}*

*## Method 3.1.2: Parallel Cross-Sectional Logistic Regression Models*

*for(i in 1:2) {*

*temp.dat1 <- dat1.1[, c(1:2, 4:7, 11:12, i+8)]*

*temp.dat1$bmi_cat_new <- as.factor(temp.dat1$bmi_cat_new)*

*temp.dat1$race_cat_new <- as.factor(temp.dat1$race_cat_new)*

*temp.dat1$edu_cat_new <- as.factor(temp.dat1$edu_cat_new)*

*for(j in 1:4) {*

*temp.dat2 <- temp.dat1[which(dat1$visit == j), ]*

*temp.mod <- glm(PRETERM ~ ., data = temp.dat2, family = binomial)*

*mod.cross[[(j+(i-1)*4)]] <- temp.mod*

*}*

*}*

*## Method 3.1.3: Model Using Mean Exposure Across Visits as a Summary*

*dat.mean <- aggregate(dat1.1[, c(1:2, 4:7, 11:12, 9:10)], list(id = dat1$id), mean)*

*dat.mean$bmi_cat_new <- as.factor(dat.mean$bmi_cat_new)*

*dat.mean$race_cat_new <- as.factor(dat.mean$race_cat_new)*

*dat.mean$edu_cat_new <- as.factor(dat.mean$edu_cat_new)*

*mod.mean <- vector("list", 2)*

*for(i in 1:2) {*

*temp.dat <- dat.mean[, c(2:9, i+9)]*

*temp.mod <- glm(PRETERM ~ ., data = temp.dat, family = binomial)*

*mod.mean[[i]] <- temp.mod*

*}*

*## Method 3.1.4: Model Using Maxmium Exposure Value Across Visits as Summary*

*for(i in 1:2) {*

*max.id <- aggregate(dat1.1[, i+8], list(id = dat1$id), which.max)*

*for(j in 1:length(unique(dat1.1$id))) {*

*max.ind[j] <- which(dat1.1$id == unique(dat1.1$id)[j])[max.id[j, 2]]*

*}*

*temp.dat <- dat1.1[max.ind, c(1:2, 4:7, 11:12, i+8)]*

*temp.dat$bmi_cat_new <- as.factor(temp.dat$bmi_cat_new)*

*temp.dat$race_cat_new <- as.factor(temp.dat$race_cat_new)*

*temp.dat$edu_cat_new <- as.factor(temp.dat$edu_cat_new)*

*temp.mod <- glm(PRETERM ~ ., data = temp.dat, family = binomial)*

*mod.max[[i]] <- temp.mod*

*}*

*## Method 3.2.1: Two Stage Mixed-effects Model*

*require(lme4)*

*pred.int <- matrix(0, length(unique(dat$id)), 2)*

*temp.dat <- dat1.1[, c(3, 8:9)]*

*colnames(temp.dat)[3] <- "X"*

*temp.mod <- lmer(X ~ gat + (gat | id), data = temp.dat)*

*pred.int[, 1] <- ranef(temp.mod)$id[, 1]*

*pred.slope <- ranef(temp.mod)$id[, 2]*

*mod.twostage1 <- vector("list", 2)*

*temp.dat <- cbind(dat.mean[, 2:9], fitted.int = pred.int[, 1], fitted.slope = pred.slope)*

*temp.mod <- glm(PRETERM ~ ., data = temp.dat, family = binomial)*

*mod.twostage1[[1]] <- temp.mod*

*## Method 3.2.2: Generalized Additive Mixed Model to Contrast Exposure Trajectories require(gamm4)*

*mod.gamm <- vector("list", 2)*

*for(i in 1:2) {*

*temp.dat <- dat1.1[, c(1:8, 11:12, i+8)]*

*colnames(temp.dat)[11] <- "Y"*

*temp.dat$bmi_cat_new <- as.factor(temp.dat$bmi_cat_new)*

*temp.dat$race_cat_new <- as.factor(temp.dat$race_cat_new)*

*temp.dat$edu_cat_new <- as.factor(temp.dat$edu_cat_new)*

*mod.gamm[[i]] <- gamm4(Y ~ s(gat) + s(gat, by = PRETERM) + age + race_cat_new + insur_new +*

*edu_cat_new + bmi_cat_new + sg + urineam, data = temp.dat, random = ~ (1 | id))*

*}*

*## Method 3.3.1: Gaussian Mixture Model by Clustering the Exposure Values*

*require(mclust)*

*mod.mclust <- vector("list", 2); pval <- rep(0, 2)*

*for(i in 1:2) {*

*temp.dat <- reshape(data.frame(dat1.1[, c(1:8, i+8, 11:13)]),*

*timevar = "visit", idvar = "id", direction = "wide")*

*temp.dat1 <- temp.dat[complete.cases(temp.dat), ]*

*sgavg2 <- sgavg[complete.cases(temp.dat)]*

*temp.mod1 <- Mclust(temp.dat1[, c(9, 19, 29, 39)], G = 2:9)*

*cluster <- temp.mod1$class*

*temp.dat2 <- cbind(temp.dat1[, 2:7], sgavg2, urineam = temp.dat1[, 11], cluster)*

*temp.dat2$bmi_cat_new.1 <- as.factor(temp.dat2$bmi_cat_new.1)*

*temp.dat2$race_cat_new.1 <- as.factor(temp.dat2$race_cat_new.1)*

*temp.dat2$edu_cat_new.1 <- as.factor(temp.dat2$edu_cat_new.1)*

*temp.dat2$cluster <- as.factor(temp.dat2$cluster)*

*temp.mod2 <- glm(PRETERM.1 ~., data = temp.dat2, family = binomial)*

*mod.mclust[[i]] <- temp.mod2*

*}*

*## Method 3.3.2: Functional Clustering Model*

*require(fda)*

*mod.fclust <- vector("list", 2)*

*for(i in 1:2) {*

*temp.dat <- reshape(data.frame(dat1.1[, c(1:8, i+8, 11:13)]),*

*timevar = "visit", idvar = "id", direction = "wide")*

*mat.y <- temp.dat[, c(9, 19, 29, 39)]*

*mat.arg <- temp.dat[, c(8, 18, 28, 38)]*

*bas <- create.bspline.basis(range(mat.arg, na.rm = TRUE), nbasis = 10)*

*coef.mat <- matrix(0, 10, dim(mat.arg)[1])*

*for(j in 1:dim(mat.arg)[1]) {*

*x.temp <- as.numeric(mat.arg[j, ])*

*y.temp <- as.numeric(mat.y[j, ])*

*mis.x <- which(is.na(x.temp)==1)*

*mis.y <- which(is.na(y.temp)==1)*

*rm.xy <- union(mis.x, mis.y)*

*x.temp <- x.temp[setdiff(1:4, rm.xy)]*

*y.temp <- y.temp[setdiff(1:4, rm.xy)]*

*if(length(x.temp)>1) coef.mat[, j] <- Data2fd(x.temp, y.temp, bas)$coef*

*}*

*rm.col <- which(apply(coef.mat==0, 2, sum)==10)*

*coef.mat2 <- coef.mat[, -rm.col]*

*fd.obj <- fd(coef.mat2, bas)*

*deriv.fdobj <- deriv.fd(fd.obj, 0)*

*harmfdPar <- fdPar(deriv.fdobj)*

*fpca.obj <- pca.fd(deriv.fdobj, 10, harmfdPar)*

*km.obj <- kmeans(fpca.obj$scores, centers = 2, nstart = 10, iter.max=100)*

*sgavg3 <- sgavg[-rm.col]*

*temp.dat2 <- cbind(temp.dat[-rm.col, 2:7], sgavg3, temp.dat[-rm.col, 11], cluster = as.factor(km.obj$cluster))*

*temp.dat2$bmi_cat_new.1 <- as.factor(temp.dat2$bmi_cat_new.1)*

*temp.dat2$race_cat_new.1 <- as.factor(temp.dat2$race_cat_new.1)*

*temp.dat2$edu_cat_new.1 <- as.factor(temp.dat2$edu_cat_new.1)*

*mod.fclust[[i]] <- glm(PRETERM.1 ~., data = temp.dat2)*

*}*

*## Method 3.3.3: Functional Logistic Regression Model*

*require(fpca)*

*temp.dat1 <- reshape(data.frame(dat1.1[, c(1:8, 9, 11:13)]),*

*timevar = "visit", idvar = "id", direction = "wide")*

*temp1 <- dat1.1[, c(3, 9, 8)]*

*temp1 <- matrix(as.numeric(as.matrix(temp1[complete.cases(temp1), ])),,3)*

*rm.list1 <- which(temp1[, 1] %in% as.numeric(names(which(table(temp1[, 1])==1))))*

*temp1 <- temp1[-rm.list1, ]*

*temp1[, 3] <- 0.001 + (temp1[, 3]-min(temp1[, 3]))/(max(temp1[, 3])-min(temp1[, 3]))*0.998*

*fpca.final1 <- fpca.mle(temp1, 9, 2)*

*fscore1 <- fpca.score(temp1, fpca.final1$grid, fpca.final1$fitted_mean,*

*fpca.final1$eigenvalues, fpca.final1$eigenfunctions, fpca.final1$error_var,*

*K = 2)*

*dat.mean <- aggregate(dat1.1[, c(1:2, 4:7, 11:12, 9:10)], list(id = dat1$id), mean)*

*dat.mean$bmi_cat_new <- as.factor(dat.mean$bmi_cat_new)*

*dat.mean$race_cat_new <- as.factor(dat.mean$race_cat_new)*

*dat.mean$edu_cat_new <- as.factor(dat.mean$edu_cat_new)*

*dat.mean <- dat.mean[which(dat.mean[, 1] %in% temp1[, 1]), ]*

*temp.dat <- cbind(dat.mean[, 2:9], fscore1)*

*temp.mod <- glm(PRETERM ~ ., data = temp.dat, family = binomial)*

***##*** *Bayesian Approach (Warren et al. 2012)*

**The codes are modified from the*[*web-based supplementary materials*](http://onlinelibrary.wiley.com/store/10.1111/j.1541-0420.2012.01774.x/asset/supinfo/BIOM_1774_sm_suppmat.pdf?v=1&s=2abbc9476e5bbcd106ec1ab656aca549cb95309a)

*library(msm) library(mnormt)*

*dat.fin <- temp.dat2[complete.cases(temp.dat2), ]*

*z <- as.matrix(dat.fin[, 9:12])*

*temp.mod <- lm(PRETERM.1 ~ age.1 + race_cat_new.1 + insur_new.1 + edu_cat_new.1 + bmi_cat_new.1 + sgavg +*

*urineamavg, data = dat.fin)*

*x <- model.matrix(temp.mod)[, -1]*

*N <- dim(x)[1]*

*M <- 4*

*Y <- dat.fin$PRETERM.1*

*corr_fun <- function(phi1_trans_val){*

*phi1_val<-(b_phi1*exp(phi1_trans_val) + a_phi1)/(exp(phi1_trans_val) + 1)*

*times <- 1:M*

*rho <- exp(-phi1_val)*

*H <- abs(outer(times, times, "-"))*

*K <- rho^H*

*p <- nrow(K)*

*K[cbind(1:p, 1:p)] <- K[cbind(1:p, 1:p)]*

*corr_inv <- chol2inv(chol(K))*

*logdeter <- log(det(K))*

*return(list(corr_inv=corr_inv,logdeter=logdeter))*

*}*

*phis_trans_log_full_cond_fun <-function(phi1_trans_val){*

*val<- -(1/2)*corr_info[[2]] - (1/(2*phi0[i]))*t(theta[i,])%*%corr_info[[1]]%*%theta[i,] + phi1_trans_val - 2*log(1+exp(phi1_trans_val))*

*return(val)*

*}*

*samples <-10000*

*Ratio.all <- first.all <- second.all <- rep(0, samples)*

*beta <- matrix(0,nrow=samples,ncol=ncol(x))*

*theta <- matrix(0,nrow=samples,ncol=ncol(z))*

*theta[1, ] <- rep(0, 4)*

*phi0 <- rep(0,times=samples)*

*phi1_trans <- rep(0,times=samples)*

*phi2_trans <- rep(0,times=samples)*

*neg_two_loglike <- rep(0,times=samples)*

*a_phi1 <- 0.2 # Lower bound for the phi1 uniform prior*

*b_phi1 <- 5 # Upper bound for the phi1 uniform prior*

*sigma2_beta <- 1000000000 # Prior variance for beta parameters*

*phi0[1] <- 0.45*

*phi1_trans[1] <- log((2-a_phi1)/(b_phi1-2))*

*xtx <- t(x)%*%x*

*xtz <- t(x)%*%z*

*ztz <- t(z)%*%z*

*ztx <- t(z)%*%x*

*var1<- chol2inv(chol(xtx + diag((1/sigma2_beta),nrow=nrow(xtx),ncol=ncol(xtx))))*

*acctot <- 0*

*mhvar_phi1 <- 10*

*corr_info <- corr_fun(phi1_trans[1])*

*for(i in 2:samples){*

*w <- (((1-Y)*rtnorm(n=N,mean=x%*%beta[(i-1),]+z%*%theta[(i-1),], sd=1, lower=-Inf, upper=0))+ ((Y)*rtnorm(n=N,mean=x%*%beta[(i-1),]+z%*%theta[(i-1),],sd=1, lower=0, upper=Inf)))*

*beta[i,] <- rmnorm(n=1,mean=((var1)%*%(t(x)%*%w-(xtz%*%theta[(i-1),]))),(var1))*

*var2 <- chol2inv(chol((ztz)+((1/phi0[i-1])*corr_info[[1]])))*

*theta[i,] <- rmnorm(n=1,mean=((var2)%*%(t(z)%*%w-(ztx%*%beta[i,]))),(var2))*

*phi0[i] <- rgamma(n=1,shape=((M/2)+3), rate=(((t(theta[i,])%*%(corr_info[[1]])%*%theta[i,])/2)+1))*

*phi0[i] <- 1/phi0[i]*

*corr_info_old <- corr_info*

*phi1_trans[i] <- phi1_trans[i-1] + rnorm(n=1,mean=0,sd=sqrt(mhvar_phi1))*

*second <- phis_trans_log_full_cond_fun(phi1_trans[i-1])*

*corr_info <- corr_fun(phi1_trans[i])*

*first <- phis_trans_log_full_cond_fun(phi1_trans[i])*

*Ratio <- exp(first-second)*

*Ratio.all[i] <- Ratio*

*first.all[i] <- first*

*second.all[i] <- second*

*if(Ratio >= 1){acc <- 1}*

*if(Ratio < 1){*

*if(Ratio < runif(n=1,min=0,max=1)){*

*phi1_trans[i] <- phi1_trans[i-1]*

*corr_info <- corr_info_old*

*acc<-0*

*}*

*else{acc<-1}*

*}*

*print(acctot/i)*

*print(c(i/samples,"2 Uniform Priors, No Space"))*

*}*
